# Risks Associated with the Use of Garcinia as a Nutritional Complement to Lose Weight

**DOI:** 10.3390/nu13020450

**Published:** 2021-01-29

**Authors:** Naroa Andueza, Rosa M. Giner, Maria P. Portillo

**Affiliations:** 1 Nutrition and Obesity Group, Department of Pharmacy and Food Science, Lucio Lascaray Research Institute, University of the Basque Country (UPV/EHU), 01006 Vitoria, Spain; naroaandueza97@gmail.com; 2 Department of Pharmacology, Faculty of Pharmacy, University of Valencia, 46100 Burjassot, Spain; Rosa.M.Giner@uv.es; 3 Spanish Agency of Food Safety and Nutrition (AESAN), 28014 Madrid, Spain; 4 Bioaraba Health Research Institute, 01009 Vitoria, Spain; 5 CIBEROBN Physiopathology of Obesity and Nutrition, Institute of Health Carlos III, 01006 Vitoria, Spain

**Keywords:** *Garcinia cambogia*, nutritional supplements, weight loss, hepatotoxicity, serotonin toxicity

## Abstract

Nowadays, obesity is one of the great nutritional problems facing public health. The prevalence of this pathology has increased in a worrying way over recent years, currently reaching epidemic proportions. In this context, nutritional supplements are presented as a therapeutic alternative to which more and more people are turning to. Nutritional supplements to lose weight based on the Garcinia plant, specifically on *Garcinia cambogia*, are commonly used. The active principle of this plant to which these properties have been attributed, is hydroxycitric acid (HCA). The aim of the present review is to gather reported data concerning the effectiveness of nutritional supplements based on Garcinia extracts on weight loss and their possible negative effects. Contradictory results have been observed regarding the effectiveness of the supplements. While statistically significant weight loss was observed in some studies, no changes were found in others. Regarding safety, although Garcinia supplements have been revealed as safe in the vast majority of the studies carried out in animal models and humans, some cases of hepatotoxicity, serotonin toxicity and mania have been reported. In conclusion, the results suggest that Garcinia-based supplements could be effective in short-term weight loss, although the data are not conclusive. In addition, the safety of the complement should be further studied.

## 1. Introduction

Obesity is one of the most common nutritional problems worldwide, currently reaching epidemic proportions. This pathology was previously considered typical in developed countries, but nowadays it also shows a high prevalence in underdeveloped countries. According to figures from the World Health Organization (WHO), in 2019 more than 1.9 billion adults aged ≥18 years were overweight, and of them more than 650 million were obese [1]. Since 1975 the worldwide prevalence of obesity has tripled, thus leading to a major public health problem. Obesity is associated with a large number of comorbidities, such as type 2 diabetes mellitus, hypertension, dyslipidemia, non-alcoholic fatty liver disease and cardiovascular diseases, among others. Furthermore, according to the WHO, people with obesity have a 50 to 150% increased risk of death from any cause compared to individuals with normal weight [2].

Hypocaloric diets and physical activity based treatments for overweight and obesity represent the first line of therapy. Due to the difficulty in achieving and maintaining an adequate adherence to this treatment, many people often turn to nutritional supplements that promise to help them lose weight in the short run or at least, to maintain it. For many people it is an easy solution, which enables them not to modify their lifestyle too much. In many instances, the motivation for the use of these supplements is due to aesthetic reasons, since the beauty standards that mark our society today encompass being slim.

These supplements work through five basic mechanisms, these being stimulation of thermogenesis, reduction in lipogenesis, increase in lipolysis, suppression of appetite, and decrease in lipid absorption. Among these nutritional supplements we find those based on plant extracts, which have been used for many centuries in the Eastern world. Nowadays, their use has become more and more prevalent throughout the world. Among them, *Garcinia cambogia* is one of the most promoted as a potential anti-obesity agent and has received a lot of attention in the media [3].

Although the current consumption of herbs and dietary supplements is unknown, in a study carried out in six European countries, it was estimated that 18.8% of the 2358 consumers selected for the study consumed one or more dietary supplements, not taking into account herbal products. The percentages of plant food supplement consumers were 9.6% in Finland, 16.9% in Germany, 22.7% in Italy, 17.9% in Romania, 18.0% in Spain and 19.1% in the United Kingdom [4].

**Figure 1:**
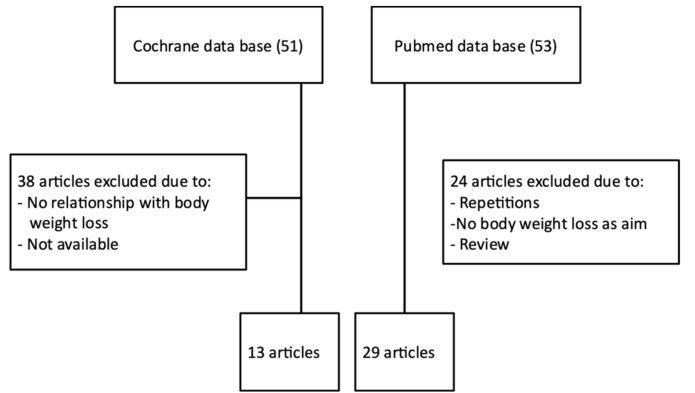
Flow chart showing the process for the inclusions of articles.

The aim of this review is to gather the reported information concerning both the effectiveness and the side-effects of nutritional supplements based on *Garcinia cambogia* to promote weight loss. In addition, the efficacy of other *Garcinia* species is also presented. For this purpose, a selective literature search in PubMed and Cochrane databases was performed. To search for the effects on body weight loss, the terms Garcinia, HCA, weight loss, fat mass and obesity were introduced, adding “and”, “or”, “not” to improve the classification. In addition, the filters “clinical trial” and “meta-analysis” were used. To search for toxic effects, the terms Garcinia, HCA and toxic effects were used. In the Cochrane Library database we sought the term “Garcinia” in the title or the abstract in meta-analyses and clinical trials. The search was extended to a total of 25 years (Figure 1).

## 2. Active Principle and Mechanisms of Action

**Figure 2:**
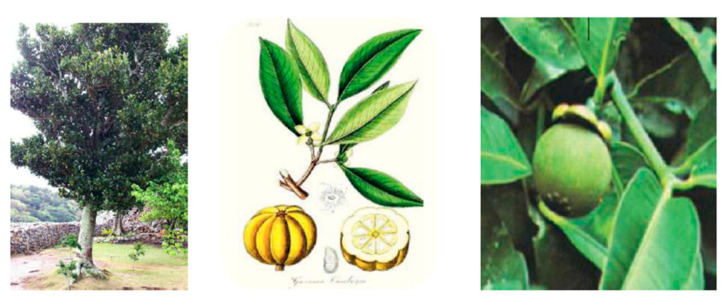
Tree, branch, and fruit of *Garcinia cambogia*.

The genus *Garcinia,* native to Asia and Africa, belongs to the *Clusiaceae* family and includes more than 300 species, such as *Garcinia cambogia* (Figure 2)*, Garcinia mangostana* and *Garcinia atroviridis* [5]. Potential therapeutic effects, such as anti-obesity, anti-ulcerogenic, antioxidant, anti-diabetes, anti-fungal, anti-inflammatory and anti-neoplasic [6], have been attributed to this genus. This has led to multiple investigations by pharmaceutical companies [5]. Some of the bioactive compounds isolated from Garcinia are garcinol, isogarcin, (−)-hydroxycitric acid (HCA), mangostin, and xanthoquimol [6]. It should be noted that out of the species mentioned above, *Garcina cambogia* is the most used as a nutritional supplement for weight loss or maintenance. The anti-obesity properties have been attributed to HCA, which is present in the rind or epicarp of the fruit and represents 20–30% of the dry weight [7]. Many food supplements containing HCA are currently marketed for weight reduction.

**Figure 3:**
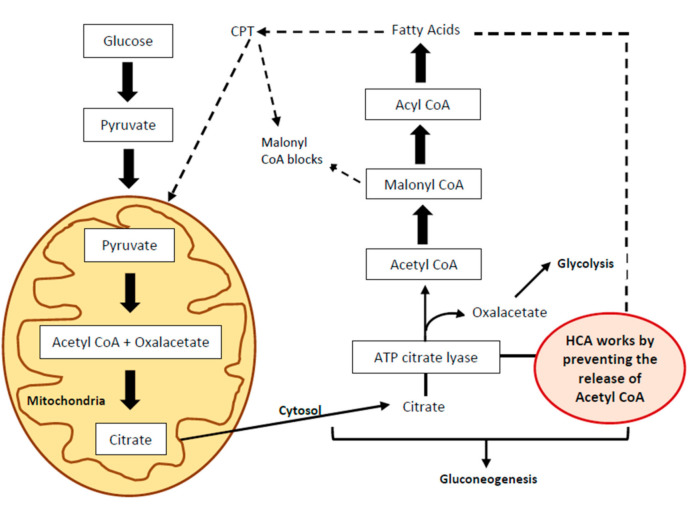
Mechanisms of action of hydroxycitric acid (HCA). CPT: Carnitine palmitoyltransferase.

The effects of HCA are associated with a reduction in food intake via serotonin level regulation and metabolic modifications, such as an increase in fat oxidation, a decrease in de novo lipogenesis and the stimulation of hepatic glycogenesis, thus promoting energy expenditure. HCA is a competitive inhibitor of adenosine triphosphate (ATP)-citrate lyase, an enzyme that catalyzes the extramitochondrial breakdown of citrate into oxalacetate and acetyl-CoA, thus limiting the availability of acetyl-CoA, a compound that plays a key role in the synthesis of fatty acids in diets rich in carbohydrates (Figure 3).

## 3. Effectiveness of Garcinia to Lose and Maintain Body Weight

In in vitro studies, HCA has been shown to inhibit fatty acid synthesis [8]. Specifically, in isolated hepatocytes, HCA inhibits the synthesis of fatty acids from glucose, but not from acetate. Therefore, HCA is an inhibitor of lipogenesis only if cytoplasmic acetyl-CoA is produced by ATP-citrate lyase. Nevertheless, fatty acid synthesis is able to continue as long as acetate, another acetyl-CoA precursor, is available. As the synthesis of acetyl-CoA is reduced, that of malonyl-CoA is reduced too, thus decreasing the negative feedback of carnitine acyltransferase. This produces an increase in lipid transport in the mitochondria and inefficient oxidation, that promotes the formation of ketone bodies. These molecules can pass into the bloodstream and reach the brain, where they constitute an energy reserve in the event of fasting [8].

In preclinical studies using animal models, chronic oral administration of HCA to rats significantly reduces food intake in the first hour after administration, together with body weight and concentrations of cholesterol, triglycerides and fatty acids. When evaluating the acute and chronic effects of HCA on energy metabolism in mice, it was observed that oral administration of 10 mg increased serum concentration of free fatty acids and glycogen concentration in skeletal muscle [9].

Leonhardt et al. (2002) studied the long-term effect of HCA in male Sprague–Dawley rats after a notable body weight loss in two different experiments. Each experiment had 23 or 24 rats, respectively [10]. The rats were fed a 1% fat diet or a 12% fat diet, depending on the experiment. Both diets were supplemented with 3% HCA. HCA produced a long-term reduction in body weight recovery in both groups (in both rats fed 1% fat or 12% fat diets). However, only HCA produced a long-term suppressive effect in the case of the group of rats fed the 12% fat diet. No effect on plasma β-hydroxybutyrate levels was observed, so the hypothesis that increased fatty acid oxidation in the liver is involved in suppressing food intake by HCA was not supported by these results [11]. In another study, young lean and obese female Zucker rats were fed a diet (70% glucose, 1% corn oil) supplemented with HCA (52.6 mmol/kg diet) for 39 days. Amongst the lean rats, HCA decreased food intake, body weight, the percent of body fat, and fat cell size. Amongst the obese rats, food intake and body weight were lowered, but body fat percentage remained unchanged [10].

In other studies, instead of isolated HCA, extracts of Garcinia were used for supplementation. Saito et al. (2005) studied the ability of a *Garcinia cambogia* extract, containing HCA, to suppress the accumulation of body fat in growing male obese Zucker rats (6 weeks) [12]. They were given diets containing different amounts of the extract, which provided 10, 51, 102 or 154 mmol of HCA/kg of diet, respectively, for 92–93 days. Rats fed the highest amount of extract, and therefore receiving the highest dose of HCA (154 mmol HCA/kg diet), significantly decreased epididymal fat accumulation, compared to the other groups.

Table 1 presents a summary of the results obtained in 20 reported intervention studies in humans [13,14,15,16,17,18,19,20,21,22,23,24,25,26,27,28,29,30,31,32]. In 12 studies, statistically significant reductions in body weight were observed [14,15,18,19,20,21,22,26,27,28,30,31]. Conversely, the supplementation turned out to be ineffective in the remaining studies [13,16,17,23,24,25,29,32]. In the studies where significant weight loss was observed, the average value was between 2 and 6 kg in 2–4 months, with the exception of one where a loss of 14 kg was induced in 6 months [31]. Interestingly, in 11 studies [19,20,21,23,24,26,27,29,30,31,32], significant reductions in other parameters, such as waist and hip circumference, triglycerides, cholesterol and glucose were also observed regardless of body weight loss. It was specified in the vast majority of the studies that the supplement should be consumed approximately half an hour before meals. Splitting the doses of HCA has also been shown to be more effective than utilizing the same amount given as a single dose.

Of note, important differences in the experimental design can be observed among the reported studies. The number of participants ranged from 11 to 214. The fact that the majority of the studies had a small sample size limits the reliability of the results. In addition, in some cases there is a lack of proportion between men and women. The inclusion of both genders is important in order to determine whether there is sexual dimorphism in the effectiveness of the nutritional supplement. Conversely, in all the selected studies, the participants were overweight or obese (based on body mass index) and the duration of treatments ranged from 2 to 4 months in the majority studies, with the exception of two longer ones, in which the duration was 6 or 6.5 months.

The variability is also observed in the type of supplement administered. Upon that, *Garcinia cambogia*/HCA was used in 15 studies, *Garcinia mangostana* in four studies and *Garcinia artroviridis* in one study. Lastly, in some cases HCA or Garcinia appeared to be combined with other ingredients, such as glucomannan (fiber), *Sphaeranthus indicus* or *Coffea arabica*. Consequently, it is not possible to determine whether the effect produced was due to the HCA content, to other ingredients, or to a combination of the effects of the different ingredients. The dose administered is another differential aspect among the studies. Last of all, the presentation of the supplement is different (pills, capsules or sachets). It is indicated in all cases that the supplement should be consumed before the meal (approximately half an hour before).

Differences in other aspects of the experimental design, such as diet and physical activity can also be found. In some studies, the participants were instructed to continue with their usual diet and physical activity, while in others they were given specific instructions. Lastly, in others they were prescribed a hypocaloric diet along with specific practice of regular physical activity.

Some of these differential aspects could explain the lack of effect observed in several studies, for instance, the lowest sample size in the studies reported by Kovacs et al. (2001) [16], Kovacs et al. (2001) [17] and Watanabe et al. (2018) [32] In the study reported by Heymsfield et al. (1998) [13], *Garcinia cambogia* was not effective since the diet was low in energy and high in fiber. The amount of fiber could have inhibited the gastrointestinal absorption of HCA (active compound in *Garcinia cambogia*) and the low energy supplied (approximately 1200 Kcal) could have affected the usefulness of HCA. In addition, the dose of HCA was lower than that used in other studies. Another parameter that could have an influence was the excess of calcium used to stabilize HCA, that could have reduced the solubility of this bioactive compound, and therefore its bioavailability [8]. In a similar way, the lack of effect in the studies carried out by Roongpisuthipong et al. (2007) [21] and Vasques et al. (2014) [29] could be related to the low amount of energy provided by the diet (1000 Kcal/d and 1500 Kcal/d, respectively). In the study conducted by Vasques et al. (2008) [23], the only apparent potential reason could be that neither dieting, nor practicing physical activity were recommended. In contrast, in all the studies in which positive results were obtained, a healthy lifestyle was either prescribed or recommended. Last of all, in the studies conducted by Kim et al. (2011) [24] and Hayamizu et al. (2003) [18], in an Asian population, it is believed that the fact that the fruit of the Garcinia is of common use as part of the traditional diet, could have led to a reduced susceptibility to its effects.

Onakpoya et al. (2011) published a systematic review and meta-analysis of randomized clinical trials devoted to evaluating the effectiveness of Garcinia extracts as weight reduction agents [33]. The authors concluded that these extracts generated short-term weight loss. However, the scale of this effect was small, since it was not statistically significant when only rigorous randomized clinical trials were considered. Therefore, the clinical relevance of these products appears to be questionable and they do not represent an altogether effective measure of the treatment of overweight and obesity.

## 4. Negative Effects on Health

### 4.1. Animal Toxicity Studies

Studies of acute, short-term, sub-chronic, and chronic toxicity, as well as studies of genotoxicity, cytotoxicity and toxicity in reproduction, have been conducted in different animal species, although mainly in rats and rabbits. These studies have shown that Garcinia/HCA have good safety profiles, so that they may be used as nutritional supplements for the treatment of obesity [34,35,36].

Ohia et al. (2002) evaluated the effects of Super Citri-Max™, a novel calcium/potassium-HCA extract (HCA-SX), containing 60% HCA, administered for 14 days. This extract is considerably more soluble and bioavailable than calcium-based HCA ingredients [37]. The study was conducted in Albino rats (males and females) fed ad libitum, treated with a dose of 5000 mg/kg (through a gastric probe), which is equivalent to 350 g or 233 times the maximum dose of 1.5 g/day of HCA in humans. The authors did not report any death or significant clinical changes. Furthermore, no significant tissue injuries were observed during the necropsy, which led them to suggest that the LD50 oral administration of HCA-SX in rats was over 5000 mg/kg. Similarly, in another study carried out by the Wil Research Laboratories, it was shown that 5000 mg HCA/kg of body weight did not produce visible symptoms of toxicity or death in animal models. In line with these studies, Clouatre et al. (2013) defended that HCA was extremely safe and this was corroborated by various reviews where it was claimed that HCA from *Garcinia cambogia* had a protective effect on the liver [38].

Shara et al. (2003) analyzed the effects of HCA intake on weight, testicular and liver lipid peroxidation, and DNA fragmentation, in addition to possible histopathological changes in Sprague–Dawley rats [39]. The animals received 0.2, 2.0, or 5.0% HCA (100–2500 mg/kg) in their diet, equivalent to approximately 100, 1000, and 2500 mg/kg/day, respectively, in humans. The lowest dose was equivalent to the daily recommended dosage in humans, but the doses of 2.0 and 5.0% are 10 to 25 times higher doses. The rats were euthanized at 30, 60, and 90 days of treatment. After 90 days of HCA administration, rats showed decreased body weight, without changes in liver or testicular lipid peroxidation or in DNA fragmentation In a follow-up study, the same authors did not find differences in the weight of various organs. Moreover, no haematological or biochemical disorders or significant histopathological changes or mortality differences were found [40].

As an exception, Kim et al. (2013) in a study addressed in rats fed a high-fat diet (45% of total energy), it was shown that after 16 weeks of treatment with *Garcinia cambogia* (1%, *w/w*, 60% HCA) oxidative stress, inflammation and liver fibrosis were triggered [41]. Consequently, it appears that the form of HCA regarding its extraction process and the residual compounds, among other factors, may spur differences between study outcomes [42].

Toxicological studies have been also addressed with other Garcinia species. Farombi et al. (2013) carried out a study in adult male Wistar rats randomly assigned to four groups of 10 rats each group given *Garcinia kola* orally at different doses (0, 250, 500 and 1000 mg/kg) for 6 weeks [43]. After conducting the study, it was concluded that the administration of *Garcinia kola* increased the antioxidant status and did not produce adverse effects on the liver, testicles and sperm. Saiyed et al. (2015) performed various toxicological studies both in vitro and in animals to evaluate the safety of Meratrim™, a supplement that contains *Garcinia mangostana* [44]. Meratrim™ was determined to be non-irritating to the skin, mildly irritating to the eyes, not mutagenic, and the no-observed-adverse-effect level (NOAEL) for this supplement was 1000 mg/kg body weight/day (in Sprague–Dawley rats). The authors concluded that the safety of Meratrim was demonstrated given the results observed in this study, added to the clinical trials of tolerability already carried out.

### 4.2. Clinical Toxicity

Based on toxicological studies, Soni et al. (2004) noted that there was sufficient quantitative and qualitative scientific evidence, from both animal and human data, to suggest that HCA intake up to 2800 mg/day is safe for human consumption [35]. As a result, a NOAEL of 2800 mg/d was established [34,35].

Accordingly, none of the studies included in Table 1, devoted to analyzing the effects of Garcinia on body weight reductions at doses below this value have found serious adverse effects. Some of the minor side effects observed were leg cramps, heartburn, diarrhoea, increased gas, higher appetite, headaches, heartburn, rash, menstrual bleeding, and general weakness. In spite of this, other authors have reported toxic manifestations, including hepatotoxicity, acute pancreatitis, serotonin toxicity and psychosis after the consumption of Garcinia-containing products (either as an extract containing other components or pure). In 16 of the 21 cases described in Table 2 [45,46,47,48,49,50,51,52,53,54,55,56,57,58,59,60], these adverse effects have occurred after the intake of formulations which contained other ingredients besides Garcinia. One of these formulations is Hydroxycut™. Fourteen different products are marketed under this name, but only eight of them contain *Garcinia cambogia*. In addition, all of them are polyherbal products that can contain up to 20 different substances [34]. When toxic effects are induced by supplements that, in addition to Garcinia, contain other components, it has not been possible to confirm that Garcinia is the agent responsible for the side-effects. In spite of this, the FDA (US Food and Drug Administration) issued a warning in 2009 on Hydroxycut™ products related to hepatotoxicity, which led to the recall of these products.

The FDA warned consumers about the serious adverse effects associated with the consumption of Hydroxycut™. This recommendation was based on 23 cases of liver damage, including one death and a liver transplant. All this led to the withdrawal of the supplement from the market. Later on, García-Cortés et al. (2016) reported 29 cases of liver damage induced by this supplement [61].

Taking this into account, as it can observed in Table 2, hepatotoxic effects have also been reported when using pure *Garcinia cambogia* extracts [49,50,54] or supplements that only contained *Garcinia cambogia* with minerals or vitamins [53,55,56]. The reason that justifies the occurrence of hepatotoxic effects in these cases, but not in all the studies gathered in Table 1, is not clear. Genetic interindividual variability leading to different susceptibility to the action of Garcinia cannot be ruled out. Nevertheless, it is important to emphasize that counting all the subjects that participated in the studies described in Table 1, the lack of toxic effects refers to a quite big sample, whereas toxic effects have only been described in a reduced number of subjects.

The common pattern of symptoms, observed in all these cases, consisted of abdominal pain (predominantly in the right upper quadrant), vomiting, nausea, fatigue and alterations in liver parameters such as transaminases, alkaline phosphatase and bilirubin. Serological tests were performed to rule out other possible causes of liver damage and/or infection, such as hepatitis, Epstein–Barr virus, cytomegalovirus, etc. In eight cases the CIOMS/RUCAM scale was used. This scale is a scoring system used to establish the etiology of drug-induced liver damage, and depending on the score obtained, the substance is classified as a highly probable cause (≥9), probable cause (6–8), possible cause (3–5), unlike cause (1–2) or excluded cause (0) of liver injury. The scores obtained for the supplements in these studies ranged from 6 to 11 points; in other words, probable to highly probable cause. Importantly, after Garcinia supplement withdrawal, the symptoms subsided and all the altered parameters returned to normal levels, although in four cases the patient finally required liver transplantation.

Regarding acute pancreatitis, one case has been reported in an 82-year-old man with past medical history of obesity and two previous episodes of acute pancreatitis in the past. He denied any alcohol use and reported no recent changes in his medications, as well as the intake of *Garcinia cambogia* recently as an appetite suppressant. He was treated with bowel rest and intravenous fluid hydration, providing a significant improvement in his symptoms [62].

Other adverse effects associated with *Garcinia cambogia* ingestion are mania and psychosis, as shown in Table 3 [63,64,65,66]. Nevertheless, currently the existing scientific evidence is limited and a causal association has not yet been established with certainty. In some cases, the participants had a previous psychiatric history and/or were treated with selective serotonin reuptake inhibitors (SSRI). HCA acts as a selective serotonin reuptake inhibitor, thus increasing serotonin levels and increasing the risk of toxicity due to this neurotransmitter [63,66]. The most relevant symptoms were irritability and agitation. After the withdrawal of the supplement, the symptoms remitted, and all the altered parameters returned to normal levels.

To conclude, another type of toxicity associated with the consumption of *Garcinia cambogia* can be observed in patients with pre-existing metabolic disorders. In this line, Bystrak et al. (2017) described the case of a 56-year-old insulin-dependent, hypertensive woman with chronic hepatitis C, who developed diabetic ketoacidosis, pancreatitis, and cardiomyopathic stress after consuming a *Garcinia cambogia* supplement (1400 mg HCA/day) to lose weight [67]. Applying the algorithm described by Naranjo et al. (1981) [68] to estimate the causality of an adverse drug reaction, a value of five was obtained, meaning a probable adverse reaction to the use of *Garcinia cambogia* [67].

## 5. Concluding Remarks

The reported scientific literature shows that nutritional supplements based on Garcinia extracts are effective in just over half of the reported studies. In these cases, the supplements should not constitute a treatment per se but, they should represent complementary tools to the conventional treatment of excess body fat. Moreover, due to their positive effects on lipid and glycemic profile, these supplements could be useful for the management of the co-morbidities associated with obesity.

Garcinia-based supplements have been shown to be safe in numerous human experiments, but the growing number of cases that report significant adverse effects, mainly associated with liver damage, and to a lesser extent with serotonin toxicity and mania, may lead to reconsideration of the safety of them. Although very often HCA has been signaled as the main element responsible for the toxic effects of Garcinia supplements, it should be noted that the fruit of *Garcinia cambogia*, an important source of HCA, has been consumed for centuries in Southeast Asia and has been generally recognized as safe (GRAS) [69]. On the other hand, as explained before in this review, in many cases Garcinia supplements contain a great number of components. Consequently, the toxicity cannot be reliably attributable to Garcinia, and it is difficult to make conclusions without giving rise to doubts or objections. Furthermore, potential negative effects due to the combination of the Garcinia supplement with other dietary supplements included in the consumer diet, or even with several drugs, cannot be discarded.

Importantly, adverse effect case reports usually reflect the associations between the observed toxicity and the intake of the dietary supplement, rather than causality. These associations need to be rigorously examined and, if finally, the supplements are found to be the causative factors for the alterations observed, the true agents need to be firmly identified, along with the dose at which the negative effects are induced [69]. In this regard, an important problem in diagnosing the cause that produces the adverse effects, is that many people perceive these type of products as not harmful or as “natural” products, thus, they tend to forget to mention them when they are asked about the foods, beverages and medications that they have consumed. It is therefore likely, that there are more cases than those diagnosed, and consequently, the magnitude of the problem may be underestimated. When the dose administered or the exact content of HCA are not specified, it is not possible to identify whether the dose exceeds the value established as NOAEL. Another important aspect, is that the issue of obesity-related liver co-morbidities as a cause of liver alterations, has been poorly handled in toxic effect reports. Much more attention should be paid to obesity-associated liver diseases in the causality assessment of dietary supplements used for weight reduction [70].

In this scenario, more studies are needed to evaluate the efficacy and safety of these products, using larger sample sizes and longer follow-up periods. Finally, it should be pointed out that there are certain population groups in which the use of these supplements should be discouraged. This is the case for pregnant and lactating women. HCA can affect the production of fatty acids and cholesterol, and can directly influence the production of sterols and steroid hormones. Pregnancy is a time of extreme sensitivity to steroid hormones, therefore, these products are not recommended. In the case of children—although it has not been possible to prove that they are dangerous—the advice is not to consume them in large doses and for long periods of time. On the other hand, the evolution of patients with mild depression or with occasional episodes of hypomania who consume Garcinia should be monitored, since their situation may worsen.

## Figures and Tables

**Table 1:** Characteristics and results of published intervention studies in humans.

Reference	Type of Study	Participants	Diet and Physical Activity	Treatment	Treatment Duration	Results	
						Weight loss and Related Parameters	Other Results Observed after the Intervention
Heymsfield et al., 1998 [13]	Randomized, double-blind, placebo-controlled study.	135 subjects BMI 27–38 aged 18 to 65 years. Control: 69 Intervention: 66.	1200 Kcal/d diethigh in fibre: -Proteins: 30% -Lipids: 20% -Carbohydrates: 50% Regular physical activity	Pills: 500 mg *Garcinia cambogia* (50% HCA), 2 pills, 3 times/day	3 months	No significant differences.	
Mattes et al., 2000 [14]	Double-blind, placebo-controlled parallel group study.	89 women mean BMI of 28.6 aged 18 to 65 years. Control: 42 Intervention: 47	1200 Kcal/diet (30% lipids) Exercise was encouraged, but no formal regimen was prescribed.	Capsules: 400 mg of *Garcinia cambogia* (50% HCA), 3 times/day	3 months	Significant weight loss. Reduction in waist circumference.	
Thom et al., 2000 [15]	Randomized double-blind study	40 subjects BMI 27.5–39.0 aged ≥18 years. Control: 20 Intervention: 20.	Participants were given diet lists with advice on low-fat foods, supplying an energy intake of approximately 1200 Kcal/d, and were recommended to use this diet during the study.	Suco-Blo™ (tablets): 200 mg *Phaseolus vulgaris* extract, 200 mg inulin and 50 mg *Garcinia cambogia* extract 3 times/day.	3 months	Significant reductionin body weight andbody mass.	
Kovacs et al., 2001 [16]	Double-blind, placebo-controlled, randomized, and cross-over study.	11 obese men mean BMI of 27.4 mean age of 47 years.	Diet was divided in 3 meals without restrictions on the type and quantity of food and a maximum of one glass of alcohol drink per day.	Isoenergetic snack (cereal bar): 4 times/d Intervention 1:500 mg HCA Intervention 2:500 mg HCA + 3 g MCT	3 Intervention periods of 2 weeks separated by washout periods of 4 weeks.	No significant differences.	
Kovacs et al., 2001 [17]	Double-blind, placebo-controlled, randomized, and crossover study.	21 obese subjects mean BMI of 27 mean of age 43 years.	Diet was divided in 3 meals without restrictions on the type and quantity of food and a maximum of one glass of alcoholic drink per day.	Isoenergetic snack (cereal bar): 4 times/d Control: no supplementation Intervention 1:500 mg HCA Intervention 2:500 mg HCA + 3 g MCT	3 intervention periods of 2 weeks separated by washout periods of 2 or 6 weeks	No significant differences.	
Hayamizu et al., 2003 [18]	Double blind, randomized, placebo-controlled, parallel-group study.	44 subjects aged 20 to 65 years visceral fat area >90 cm^2^ Control: 21 Intervention: 23	Maximun 2250 Kcal/d for men and 1800 Kcal/d for women.	Tablets: 185.25 mg of *Garcinia cambogia* extract (60% HCA) 3 tablets before each meal (9 tablets/day)	3 months + 1 month of placebo in both groups at the end.	Reduction in visceral fat area, subcutaneous fat area and total fat area.	
Preuss et al., 2004 [19]	Randomized, double-blind, placebo-controlled study.	30 subjects BMI > 26 aged 21 to 50 years Control:10 Intervention 1:10 Intervention 2:10	2000 Kcal/d divided in 3 meals: - Proteins: 17% - Lipids: 25% - Carbohydrates: 58% 30 min supervised walking exercise program (5 days a week).	HCA-SX (4667 mg) divided in 3 doses: Intervention 1:2800 mg/d of HCA Intervention 2:2800 mg/d of HCA, 4 mg niacin-bound chromium and 400 mg *Gymnema sylvestre* extract.	2 months	Significant weight loss in both intervention groups. Reduction in food intake.	Reduction in total cholesterol, LDL-c, TG, and leptin levels. Increase in fat oxidation, HDL levels and serotonin levels.
Preuss et al., 2004 [20]	Randomized, double-blind, placebo-controlled study.	60 subjects BMI > 26 aged 21 to 50 years Control: 20 Intervention 1:20 Intervention 2:20	2000 Kcal/d - Proteins: 17% - Lipids: 25% - Carbohydrates: 58% 30 min supervised walking exercise program (5 days a week).	HCA-SX (4667 mg) divided in 3 doses: Intervention 1:2800 mg/d of HCA Intervention 2:2800 mg/d of HCA, 4 mg niacin-bound chromium and 400 mg *Gymnema sylvestre* extract.	2 months	Significant weight lossand reduction in food intake in both intervention groups.	Reduction in total cholesterol, LDL-c, TG and serum leptin levels in both intervention groups. Increase in HDL-c and excretion of urinary fat metabolites in both intervention groups.
Roongpisuthipong et al., 2007 [21]	Randomized, double-blind, placebo-controlled study	50 women BMI 25–30 aged 18 to 75 years Control: 25 Intervention: 25	1000 Kcal/d - Proteins: 50 g - Fats: 33 g - Carbohydrates: 125 g	Sachets: 1.15 g of *Garcinia artroviridis* (HCA) 3 times/day	2 months	Significant weight loss during the first 4 weeks. No significant differences over the following 4 weeks. Decrease in fat mass, bicipital, subscapular and suprailiac folds and upper arm circumference.	Increase in lean mass and body water. Decrease in TG.
Toromanyan et al., 2007 [22]	Double blind, randomized, parallel group, placebo-controlled study.	60 subjects BMI 25–44 aged 25 to 65 years Control: 30 Intervention: 30.	Diet and exercise performed regularly.	Slim339™ (tablets): 132 mg of *Garcinia cambogia* (HCA) + *Matricaria chamomilla, Rosa damascena, Lavandula officinalis and Cananga odorata* 3 times/d.	2 months	Significant weight reduction.	
Vasques et al., 2008 [23]	Randomized double-blind study	58 subjects BMI 30–39.9 aged 25 to 60 years Control: 26 Intervention: 32		Capsules: 800 mg of *Garcinia cambogia* (HCA) + 500 mg de *Amorphophallus konjac* → 3 times/day	3 months	No significant reduction in body weight.	Reduction in total cholesterol and LDL-c.
Kim et al., 2011 [24]	Randomized, double-blind, placebo-controlled study	86 subjects BMI 23–29 aged 20 to 60 years. Control: 29 *Glycine max* leaves (GML): 28 *Garcinia cambogia* (GC): 29	Diet and habitual physical activity	Pills: 2 g/d of the substances corresponding to each group. In the case of the placebo and GML 4 pills/d and for *Garcinia cambogia* 8 pills/day.	2.5 months	No significant reduction in body weight.	GML reduced total cholesterol and increased HDL-c (significant differences compared to the placebo group and the GC and placebo group, respectively).
Lu et al., 2012 [25]	Randomized double-blind study	114 overweight subjects	Nutritional education	Super CitriMax™ (HCA) 2800 mg/day	2 months	No significant reduction in body weight.	
Stern et al., 2013 [26]	Randomized, double-blind, placebo-controlled clinical study	60 subjects BMI 30–40 aged 21 to 50 years Control: 30 Intervention: 30	Participants were given free prepared meals. 2000 Kcal/d - Proteins: 14% - Lipids: 25% - Carbohydrates: 61% Physical activity (walking) 30 min, 5 times/d.	Capsules: 400 mg of *Sphaeranthus indicus + Garcinia mangostana*, ratio 3:1 2 times/day	2 months	Significant reduction in body weight, BMI and waist circumference.	Decrease in total cholesterol and TG and increase in adiponectin.
Stern et al., 2013 [27]	Randomized, double-blind, placebo-controlled clinical study	95 subjects BMI 30–40 aged 36 to 40 years Control: 46 Intervention: 49	Same diet and physical activityas in reference 26. In this case the diet is divided into 3 intakes.	Same treatment as in reference 26.	2 months	Reduction in body weight, BMI, waist and hip circumferences.	Decrease in total cholesterol, TG and fasting glucose. Increase in adiponectin. Improvement in physical function and self-esteem (IWQOL questionnaire).
Chong et al., 2014 [28]	Randomized, placebo-controlled, double-blind parallel group study	91 caucasian subjects BMI 25–32 aged 18 to 60 years Control: 45 Intervention: 46	Dietary advice + balanced diet with a deficit of 500 Kcal. −30% lipids.	Tablets: 850 mg 3 tablets 2 times/day. Composition: 650 mg of *Garcinia cambogia* (HCA) + 100 mg of *Camellia sinensis* + 75 mg of *Coffea arabica* + 25 mg of *Lagerstroemia speciosa*	3.5 months	Significant weight loss and reduction in BMI, body fat, waist and hip circumferences	
Vasques et al., 2014 [29]	Randomized double-blind study	43 women BMI > 25) aged 25 to 60 years Control: 13 Intervention: 30	Individualized diet, with an average caloric restriction of 1523 ± 185 Kcal/day Regular physical activity.	Capsules: 800 mg of *Garcinia cambogia* (HCA) 3 times/day	2 months	No statistically significant differences.	Reduction in TG level.
Kudiganti et al., 2016 [30]	Randomized, double-blind, placebo-controlled clinical study	60 subjects mean BMI of 28.3 aged 21 to 50 years Control: 30 Intervention: 30.	2000 Kcal/d - Proteins: 17% - Lipids: 25% - Carbohydrates: 58%	Capsules: 400 mg of Meratrim™: extracts from the flower heads of *Sphaeranthus indicus* and the fruit rinds of *Garcinia mangostana* 2 times/day	4 months	Significant weight loss and reduction in BMI, waist and hip circumferences.	Reduction in TG and LDL-c cholesterol. Increase in HDL-c.
Maia-Landim et al., 2018 [31]	Non-randomized prospective controlled intervention study	214 subjects BMI > 25 older than 18 years	Balanced diet and regular physical activity, smoking not permitted and controlof alcohol intake.	Capsules: 500 mg of *Garcinia cambogia* (HCA) + 500 mg of *Amorphophallus konjac* 2 times/day	6 months	Reduction in total fat mass and visceral fat mass after 3 and 6 months of intervention.	Increase in basal metabolic rate Reduction in glucose, total cholesterol and TG.
Watanabe et al., 2018 [32]	Prospective, randomized, controlled, parallel study	22 obese women with insulin resistance aged 18 to 65 years Control: 11 Intervention: 11	Hypocaloric diet (300 Kcal restriction) + physical activity of moderate intensity - Proteins: 20–25% - Lipids: 30% - Carbohydrates: 45–50%	Capsules: 400 mg of *Garcinia mangostana* 1 time/day	6.5 months	No significant reduction in body weight.	Reduction in insulin concentration and HOMA-IR.

BMI: body mass index; HCA: hydroxycitric acid; HOMA-IR: insulin resistance index, MCT: medium chain triglyceride; TG: triglycerides; IWQOL: impact of weight on quality of life—lite.

**Table 2:** Cases of liver damage associated with the consumption of *Garcinia cambogia* supplements in humans.

Reference	Ageyears	Sex	Type of Supplement	Duration	Symptoms	Test Performed	**Diagnosis/Type of Liver Injury**
Stevens et al., 2005 [45]	27	M	Hydroxycut^™^ 3 capsules, 3 times/d.	5 weeks	Fatigue and jaundice.	Laboratory analysis: elevated AST, ALT, AF and PT. Serological study: negative.	Hepatotoxicity Cholestatic liver injury pattern.
Stevens et al., 2005 [45]	30	M	Hydroxycut™ 9 capsules/d.	5 days	Fever, vomiting, fatigue, and jaundice.	Laboratory analysis: AST, ALT, AF and PT elevated and low albumin. Serological study: negative. CT and cholangiography: normal.	Hepatotoxicity Hepatocyte necrosis was the likely pattern of injury.
Dara et al., 2008 [46]	40	W	Hydroxycut™ 6 capsules/d.	1 week	Mid-epigastric abdominal pain, non-bloody diarrhea, fevers, chills, nausea, vomiting, anorexia and profound fatigue.	Laboratory analysis: acute hepatitis (elevated AST, ALT and AF) Serological study: negative.	Acute hepatitis.
Dara et al., 2008 [46]	33	W	Hydroxycut™	2 weeks	Nausea, crampy abdominal pain, jaundice, acholic stools, dark-colored urine, pruritus and profound fatigue.	Physical examination: jaundice and scleral icterus. Laboratory analysis: elevated AST, ALT, TB and DB. Serological study: negative.	Acute hepatitis.
Shuster et al., 2010 [47]	25	M	Exilis: *Garcinia cambogia*, *Garcinia sylvestre*, L-carnintine and chrome	3 weeks	Two weeks after starting treatment: fatigue and dark urine. In the third week: fever, nausea, vomiting and pain.	Laboratory analysis: elevated ALT, AST, TB and INRA comprehensive study was conducted to determine the etiology of liver damage, but all tests were negative.	Hepatic encephalopathy Liver transplantation required.
Sharma et al., 2010 [48]	19	M	Hydroxycut™	1 week	Fever, severe fatigue, myalgia, arthralgia, and erythematous rash over in lower extremities.	Physical examination: toxic appearance, marked jaundice and fever (39.4 °C). Laboratory analysis: elevated ALT, AF, bilirubin and PT. low blood cell count and hemoglobin. Blood culture, urinalysis, X-rays, abdominal ultrasound, CT and MRCP: normal. Serological study: negative. Hepatic biopsy: acute cholangitis.	Acute cholangitis
Mancano et al., 2015 [49]	42	W	*Garcinia cambogia* pure	1 week	Right upper quadrant abdominal pain and nausea (without emesis).	Laboratory analysis: elevated ALT, AST, AF, ferritin and INR Serological study: negative. Abdominal ultrasound: normal.	Acute hepatitis
Melendez-Rosado et al., 2015 [50]	42	W	*Garcinia cambogia* pure	1 week	Abdominal pain in the right upper quadrant, nausea without emesis and clamminess.	Laboratory analysis: elevated ALT, AST, AF and ferritin. Serological study: negative. Abdominal ultrasound: mildy coarse hepatic echotexture. CT: normal.	Acute hepatitis
Araujo et al., 2015 [51]	41	M	Hydroxycut SX-7 Clean Sensory™ 2 capsules/day 4 times/week	2 months	Malaise, jaundice, fatigue, nausea, vomiting and asterixis	Physical examination: jaundice and liver edge percussed. Laboratory analysis: elevated AST, ALT, TB, DB, PT and creatinine. Serological study: negative. Abdominal ultrasound: increased liver echogenicity and liver length. CIOMS/RUCAM: 9	Acute hepatocellular liver injury
Smith et al., 2016 [52]	26	M	Multi-ingredient protein supplement with *Garcinia cambogia* (70%).	1 week	Jaundice, fatigue and asterixis.	Laboratory analysis: elevated ALT, AST, AFand bilirubin. Serological study: negative. Hepatic biopsy: liver necrosis. CIOMS: 6	Hepatotoxicity Liver transplantation required.
Corey et al., 2016 [53]	52	W	*Garcinia cambogia* supplement: *Garcinia cambogia* extract (936 mg, 60% HCA), calcium, chromium, potassium 2 capsules/d (1000 mg/day)	3.5 weeks	Decreased appetite, worsening fatigue, and intermittent confusion.	Physical examination: abdominal distention and jaundice. Laboratory analysis: elevated ALT, AST, AF, TB, DB and INR, and low platelet count. CT: nodular liver compatible with necrosis and ascites. Serological study: negative. Biopsy: severe acute hepatitis with necrosis and parenchymal collapse. MELD: it was evolving until it reached a score of 40. CIOMS: 7	Acute liver failure. Liver transplantation required.
Lunsford et al., 2016 [54]	34	M	*Garcinia cambogia* pure 2 capsules of 80 mg, 3 times/day	6.5 months.	Nausea, vomiting, abdominal pain, and dark urine.	Laboratory analysis: elevated transaminases and bilirubin. Asterixis, jaundice, and confusion. Elevated transaminases, bilirubin, elevated INR. Images: cirrhosis or hepatocellular carcinoma. MR: no tumor process.Serological study: positive antinuclear antibody.Hepatic biopsy: necrosis with collapse of the liver architecture.	Severe liver injury. Liver transplantation required.
Crescioli et al., 2018 [55]	61	W	SUPER ANANAS SLIM™: *Garcinia cambogia* (60%), *Ananas comosus* and *Ilex paraguariensis.*	2 months	Abdominal pain, nausea, progressive weakness, jaundice, dark stools, and acholic stools.	Laboratory analysis: ALT, AST, TB, DB, albumin, AF, GGT out of normal range. Serological study: negative. Abdominal ultrasound, MRI. Doppler: normal. CT: small peritoneal effusion and perihepatic lymphadenopathy. Biopsy: cholestatic hepatitis. CIOMS: 7.	Herbal-induced liver damage.
Crescioli et al., 2018 [55]	39	W	Two sipplements: OBLESS™: *Garcinia cambogia* (72 mg of HCA) and other components: 1 capsule/day and	1 month	Jaundice, asthenia, loss of appetite, and right hypochondrial pain.	Laboratory analysis: elevated ALT, AST, TB, DB, AF, GGT, CRP and lactate dehydrogenase. Serological study: nonspecific antinuclear antibodies and positive bile antibodies. Abdominal ultrasound: normal. CIOMS: 6	Acute cholestatic
			Magistral preparation of different herbs extracts: 1 capsule/day	15 days			
Crescioli et al., 2018 [55]	47	W	THERMO GIALLO™: *Garcinia cambogia* (200 mg HCA) and chromium: y 2 capsules/da.	1 month	Severe abdominal pain.	Laboratory analysis: elevated AST, ALT and TB. Serological study: negative. CIOMS: 6.	Acute hepatitis.
Crescioli et al., 2018 [55]	52	W	2 JILL COOPER BE SLIM™: *Garcinia cambogia* (240 mg) and *Green Coffee* extract 1 capsule / d of each product	1 month		Laboratory analysis: elevated AST, ALT, BT, GGT and AF. Serological test: negative. CIOMS: 6.	Acute hepatitis.
Sharma et al., 2018 [56]	57	W	*Garcinia cambogia* (100%) and vitamin A and D supplement 2 capsules/d (2800 mg/d)	1 month	Abdominal pain (more intense in the right upper quadrant) and vomiting.	Laboratory analysis: elevated ALT, AST, TB, DB, INR, PT. Normal vitamin A and D levels. Serological study: negative Abdominal ultrasound: normal liver CIOMS/RUCAM: 11	Hepatitis secondary to the consumption of *Garcinia cambogia*. After withdrawal of the supplement the levels of the altered enzymes normalized. After six months they elevated again, coinciding with the reintroduction of the supplement.
Philips et al., 2018 [57]	33	W	Safe Lean™: *Garcinia cambogia* (600 mg), *Allium sativum* (250 mg) and *Trigonella foenum graecum* (100 mg) 1 capsule, 2 times/day	1 month	Nausea, loss of appetite	Laboratory analysis: elevated AST, ALT, AF, TB, gamma-glutamyl transferase, albumin and INR. Serological study: negative. CT: hepatomegaly. RUCAM: 8	Drug induced liver injury, secondary to Safe Lean^™^.
			Calcium, vitamin A and folic acid supplement 1 time/day	3 months			
Yousaf et al., 2019 [58]	21	W	*Garcinia cambogia* 1400 mg/day	4 weeks	Abdominal pain for 1 wk associated with nausea, vomiting, anorexia and myalgias.	Abdominal ultrasound Laboratory analysis: elevated ALT, AST, alkaline phosphatase	Hepatomegaly Acute liver failure.
Khetpal et al., 2020 [59]	22	W	Hydroxycut™ 2 capsules/day	3 months	Chest pain, fatigue, palpitations and shortness of breath	Physical examination: tachycardia, low oxygen saturation and asterixis. Laboratory analysis: elevated AST, ALT, INR, leukocytes and white blood cells. Serological study: negative. Abdominal ultrasound: hepatomegaly. RUCAM: 9.	Acute drug-induced liver injury likely due to Hydroxycut^™^.
Ferreira et al., 2020 [60]	26	W	1800 mg of *Garcinia cambogia* (900 mg HCA), 1275 mg of green tea extract with 450 mg of Veldt raisin and 1200 mg of *Coffea arabica* daily.	7 months	Fatigue, nausea and jaundice.	Laboratory analysis: acute hepatitis (elevated AST, ALT, TB and INR). Abdominal Ultrasound: normal. MRCP: normal. Transjugular liver biopsy: acute hepatitis. Serological study: negative. RUCAM: 6.	Subacute liver failure secondary to the consumption of *Garcinia cambogia*. Liver transplantation required.

M: men; W: women; ALT: alanine aminotransferase; AST: aspartate aminotransferase; AF: alkaline phosphatase; GGT: gamma-glutamyl transferase; TB: total bilirrubin; DB: direct bilirrubin; INR: international normalized ratio; PT: prothrombin time; CRP: C-reactive protein; CT: computed tomography; MRCP: magnetic resonance cholangiopancreatography; MR: magnetic resonance; CIOMS/RUCAM: scale for diagnosing drug-induced liver damage; MELD: scale to measure the severity of chronic liver disease. In cases where information about dose is not provided, it is because it was not indicated in the article.

**Table 3:** Cases of mania and serotonin toxicity associated with the intake of *Garcinia cambogia* supplements in humans.

Reference	Ageyears	Sex	Previous Psychiatric History	Type of Supplement and Treatment Duration	Psychotropic/Antidepressant Drugs	Symptoms	**Diagnosis**
Lopez et al., 2014 [63]	35	W	No	1000 mg *Garcinia cambogia* (60% HCA), chromium, potassium and calcium 2 capsules, 3 times/day. 2–3 months.	Yes: escitalopram (SSRI). 1 year.	Stuttering speech pattern, spontaneous ankle clonus, bilateral ocular clonus, rhythmic jaw movements, profuse sweating, hypertension, tachycardia, and hyperreflexia.	Serotonin toxicity associated with *Garcinia cambogia* ingestion.
Hendrickson et al., 2016 [64]	50	M	Type I bipolar disorder	*Garcinia cambogia*: 2 capsules/day. 2 months.	No: he had been stable off medications for 6 years.	Irritability, pressured speech, grandiosity, excessive spending, increased social activity and decreased need for sleep.	Bipolar I disorder, manic, severe.
	25	M	No	*Garcinia cambogia*: 1–2 capsules/day.2 months.	No	Inflated self-esteem, grandiosity, decreased need for sleep, increased activity, excessive spending, pressured speech, paranoia and religious delusions.	Bipolar I disorder, manic, severe with psychosis.
	34	W	Type II bipolar disorder and past SSRI-induced hypomania	*Garcinia cambogia* For 1–2 months.	Yes: aripiprazole, bupropion and topiramate.	Irritability, pressured speech, decreased need for sleep and agitation.	Recurrence of bipolar disorder type II, hypomania, moderate.
Cotovio et al., 2017 [65]	51	W	Type I bipolar disorder	*Garcinia cambogia*, calcium, chromium and potassium.	Yes: paroxetine (SSRI) and valproic acid	Irritability, agitation, increased energy and decreased need for sleep.	Hypomanic episode associated with ingestion of *Garcinia cambogia*.
Nguyen et al., 2019 [66]	22	W	No	*Garcinia cambogia* Plus^TM^: 500 mg *Garcinia cambogia* per capsule (60% HCA) 1 capsule/day during de first 5 days and then 3 capsules/d during the next 5 days.	No	Expansive mood, psychomotor agitation, disorganized and pressured speech, flight of ideas, grandiosity, delusions and auditory hallucinations.	Mania and psychosis secondary to *Garcinia cambogia* ingestion.

M: men; W: women; SSRI: selective serotonin reuptake inhibitor.

## Data Availability

Not applicable.
